# Clinical Findings in Children with Noonan Syndrome—A 17-Year Retrospective Study in an Oral Surgery Center

**DOI:** 10.3390/children9101486

**Published:** 2022-09-28

**Authors:** Anna Janas-Naze, Konrad Malkiewicz, Wei Zhang

**Affiliations:** 1 Department of Oral Surgery, Medical University of Lodz, 92-213 Lodz, Poland; 2 Department of Orthodontics, Medical University of Lodz, 92-213 Lodz, Poland; 3 Shanxi Oral Disease Prevention and Treatment Center, Shanxi Provincial People’s Hospital, Taiyuan 030012, China

**Keywords:** Noonan syndrome, RASopathies, RAS, genotype–phenotype correlations, oral surgery

## Abstract

To date, only a limited number of publications have studied the specific oral and maxillofacial findings in patients diagnosed with Noonan syndrome (NS), which is an example of a genetically heterogeneous RASopathy. In this retrospective study, we aimed to ascertain the genotype–phenotype correlations between genetic mutations and certain diagnoses in the field of oral surgery. We collected surgical and genetic data from 42 children (median age, 12 years) who had a confirmed diagnosis of NS and underwent surgery in the Department of Oral Surgery, Medical University of Lodz, over a 17-year period, from 2004 to 2021. In total, 17 patients with mutations of the *PTPN11* gene were diagnosed with over-retained deciduous teeth and supernumerary teeth. An amount of 7 patients with mutations of the *SOS1* gene were diagnosed with mandibular compound odontomas. Finally, 12 patients with mutations of the *LZTR1* gene were diagnosed with bilateral or unilateral central giant cell granulomas in the mandible. Although craniofacial features of many genetic disorders have been previously described in the literature, this study determined the genotype–phenotype correlations in the field of oral surgery.

## 1. Introduction

Developmental anomalies occur as a result of disturbances in the normal development of tissue, organs, or the entire body during the fetal period. Among the contributing pathogenic mechanisms, one can distinguish deformations, dysplasia, and malformations. Such anomalies constitute one of the main reasons for spontaneous abortions and infant deaths, and are simultaneously the most common cause of disabilities in children. Many developmental malformations are diagnosed only after birth, and affected children are in need of multi-disciplinary care.

Developmental anomalies can be classified as major, which significantly influence the patient’s condition and may lead to death (lethal malformations), and minor, which do not have a significant impact on the patient’s health. Minor congenital anomalies include so-called dysmorphic features, which most often concern the development, size, and position of certain craniofacial structures. In most cases, the cause of such anomalies is unknown; however, it is considered that genetic factors play a significant role. In most cases, the malformations are determined by multiple factors (genetic predisposition and environmental factors); less often, multiple congenital defects occur with a combination of major and minor anomalies, which can be random or constant. In the latter case, based on an observed combination of certain features, a specific phenotype can be ascertained [1].

The occurrence of syndromic congenital malformations is usually due to genetic changes: chromosomal aberrations of a numerical and structural nature or mutations in specific genes with a small percentage caused by impaired epigenetic regulation [2]. Many syndromes with a known genetic background have been described to date. In the case of monogenic disorders, a mutation in a specific gene leads to changes in the properties or level of protein encoded by said gene, which in turn influence cellular homeostasis, including the proper functioning of the intracellular signal-transduction pathway. Adequate activity is essential for the normal course of cellular processes, such as proliferation, growth, migration, apoptosis, or differentiation, which are pivotal for the development of tissue and organs. An example of a pathway involved in the above-mentioned processes is the RAS/mitogen-activated protein kinase (MAPK) pathway, which modifies activity and leads to the occurrence of not only developmental disorders, but also tumor growth.

Regarding organism development, normal activity of the RAS/MAPK pathway is essential for the proper growth and development of the musculoskeletal (craniofacial included) and cardiovascular (growth of endocardial cushions and valves) systems. This pathway is also involved in the progression of the nervous system (formation of dendritic spines) and synaptogenesis; consequently, its functioning is pivotal for the process of learning and memory formation; thus, alterations in its activity may lead to neurodevelopmental disorders.

Congenital anomalies associated with disturbances in the RAS/MAPK pathway are referred to as RASopathies and are associated with a variety of clinical signs and symptoms, constituting a major diagnostic challenge.

Noonan syndrome (NS) is a model example of a highly genetically heterogeneous RASopathy, meaning that the same phenotype can result from mutations within various genes; mutations in more than 14 genes have been implicated in NS [3]. *PTPN11* was the first gene in which a gain-of-function mutation was linked to NS, but additional research showed that mutations in this gene are only observed in approximately 50% of patients with a clinical diagnosis of NS [4]. Although further studies have revealed mutations in the *SOS1, RAF1, KRAS, NRAS, BRAF, SHOC2, NF1, CBL*, and *LZTR1* genes, it is estimated that approximately 25% of patients with clinical features of NS have still not been properly diagnosed [5].

NS is a disease with a highly variable clinical expression, and its course depends on the general health of the patient and the presence of comorbidities, such as cardiac defects, which may increase the risk of mortality. The Cardiac Rasopathy NETwork (CARNET) study was conducted in seven European countries, where the clinical records of 371 patients with a confirmed molecular diagnosis of RASopathy were reviewed. This study concluded that the mortality of patients with NS was 0.28 per 100 patients, and the cumulative survival rates were 98.8% and 94.3% at 1 and 20 years, respectively [6]. A higher mortality rate was observed only in patients with hypertrophic cardiomyopathy before the age of two. This relatively low mortality rate translates directly into higher life expectancy; therefore, making every effort to improve the quality of life is a must. Enhancing routine dental care comes to mind. Unfortunately, our previous experience shows that most children diagnosed with Noonan syndrome reside in orphanages or nursing homes. This study did not aim to investigate the socio-economic conditions of patients with NS treated at our center; however, long-term experience with children suffering from disabilities shows that most of them are under the care of the above-mentioned facilities. Unfortunately, such institutions may neglect the dental care of these patients in the face of more pressing medical issues. In addition, the dental management of these patients may be challenging. For example, given that a large proportion of patients diagnosed with NS are also afflicted with various hematological disorders affecting primary and secondary hemostasis [7,8] oral surgeries that involve flap elevation and osteotomy carry a high risk of hemorrhage, even in seemingly healthy subjects, as such disorders are usually not detected through routine screening.

Although genotype–phenotype correlations have been discussed by several authors previously [3,9,10], they mainly focused on ocular, cardiac, and hematological findings. Although craniofacial features have also been previously described well, genotype–phenotype correlations in the field of oral surgery have not been analyzed in detail. Hence, we attempted to determine genotype–phenotype associations with respect to oral and maxillofacial surgery, which might be helpful for clinicians in daily practice.

## 2. Materials and Methods

### 2.1. Study Design

This retrospective study focused on the medical records of patients under the age of 18, diagnosed with NS, and subjected to surgical procedures in the Department of Oral Surgery, Central Clinical Hospital of Medical University of Lodz for a period of 17 years, from 2004 to 2021. The study was approved by the Ethics Committee of the Medical University of Lodz (RNN/270/21/KE), and written informed consent was obtained from all subjects’ legal guardians. The following inclusion criteria were adopted:

- Cases with evident diagnosis of NS as asserted by previous genetic screening;
- Patients subjected to surgical procedures in the area of oral and maxillofacial surgery;
- Patients with a high-quality panoramic radiograph or cone-beam computed tomography (CBCT);
- Patients with results from histopathological examinations (if applicable).

Exclusion criteria included any prior oral surgery procedures.

### 2.2. Statistical Analysis

SPSS Statistics 25 software (IBM, Armonk, NY, USA) was used for statistical analysis.

- Spearman’s rank correlation was used to measure any statistically significant association between the variables;
- The chi-squared test was used to examine whether the equated groups had equipotential and to assess the presence of a statistically significant correlation among the nominal variables. The effect size was measured with V Cramer. For two categorical variables, the Yule phi coefficient was used;
- The Mann–Whitney U test was used to analyze statistically significant differences between two independent groups of patients;
- The Eta coefficient was used to evaluate the correlation between quantitative variables (age) and nominal variables;
- To execute the analysis, certain descriptive statistics were used: mean, median, standard deviation, minimum, maximum, and the first and third quartiles;
- A *p*-value of 0.05 or less was considered statistically significant.

## 3. Results

### 3.1. Patient Characteristics

Forty-two patients met the inclusion criteria, with the majority being females. The most common finding was the presence of over-retained deciduous teeth and supernumerary teeth (Table 1), with no statistically significant differences between sex and diagnosis (χ^2^(1) = 0.38; *p* = 0.54).

Table 2 provides descriptive statistics regarding the ages of the participants (Table 2).

Table 3 depicts the subject groups as distinguished by mutations in the genes *SOS1, RAF1, KRAS, NRAS,* and *LZTR1;* no *BRAF, SHOC2*, or *CBL* mutations were present. Only the group with *PTPN11* mutations had equipotential.

Table 4 provides an overview of the other variables analyzed. All subjects were characterized by craniofacial dysmorphia, and a statistically significant part of the group was characterized by developmental delays and cardiac defects.

### 3.2. Genotype–Phenotype Correlations

Table 5 depicts statistically significant genotype–phenotype correlations, marked in bold, and suggests that mutations of:

- *PTPN11* are correlated with over-retained deciduous teeth and supernumerary teeth;
- *SOS1* are correlated with mandibular compound odontomas;
- *KRAS* are correlated with mandibular compound odontomas;
- *LZTR1* are correlated with bilateral central giant cell granulomas in the mandible and unilateral central giant cell granulomas in the mandible.

However, the above-mentioned results should be interpreted with caution due to the small sample size. A high V Cramer coefficient suggests that a strong association is characteristic of mutations in the gene *SOS1,* although the abundance is low. In addition, a high V Cramer coefficient shows that a strong association is characteristic of mutations in the gene *LZTR1*, where higher abundance can be observed.

The prevalence of particular genetic parameters in the participants in relation to additional clinical features was also analyzed (Table 6). Four statistically significant correlations were found:

- *LZTR1* with developmental delays, χ^2^(1) = 17.01; *p* < 0.001; V_cr_ = 0.64;
- *PTPN11* with cardiac defects, χ^2^(1) = 5.06; *p* = 0.02; V_cr_ = 0.35;
- *LZTR1* with cardiac defects, χ^2^(1) = 17.01; *p* < 0.001; V_cr_ = 0.64;
- *PTPN11* with epilepsy χ^2^(1) = 8.51; *p* = 0.004; V_cr_ = 0.45.

The above-mentioned differences indicate that:

- A total of 50% of the patients with cardiac defects had a mutation of the *PTPN11* gene, while 10% of the patients with the *PTPN11* mutation had no significant cardiac findings;
- A total of 80% of patients with epilepsy had a mutation of the *PTPN11* gene, while 28.1% of patients with the *PTPN11* mutation lacked concomitant epilepsy;
- A total of 12.8% of patients with both cardiac defects and developmental delays had a mutation of the *LZTR1* gene, while 80% of patients with the *LZTR1* gene lacked cardiac defects and developmental delays, suggesting the strongest correlation.

There were no other statistically significant correlations found.

The present study also analyzed the possibility of sex affecting the analyzed data; however, no statistically significant correlations were found, as presented in Table 7.

Another analysis concerned the dependency of age on the incidence of the variables, which was analyzed using the Eta coefficient. Two statistically significant correlations were observed between *LZTR1* (*p* = 0.001) and developmental delay (*p* = 0.02), as displayed in Table 8.

The results obtained were confirmed by the observed differences. Children with developmental delays were statistically younger (Me = 11 years) than those without developmental delays (Me =14 years) (U = 45.5; *p* = 0.001).

These differences were also true for *LZTR1* (U = 14.5; *p* < 0.001). Children with such features were significantly older (Me = 14 years) than those without (Me = 11 years).

Additionally, a statistically significant correlation was observed between the two most common mutations, *LZTR1* and *PTPN11* (χ^2^(1) = 7.2; *p* = 0.01; ϕ = 0.41). In the group with mutations within *PTPN11,* a concomitant mutation within *LZTR1* was observed (5.9%). No other statistically significant correlations were observed.

## 4. Discussion

Few studies have addressed the oral and maxillofacial findings in patients diagnosed with NS. The literature search performed by Lutz et al. [11] revealed 20 published papers, most of which were case reports; one of these papers was a case series. To the best of our knowledge, there have been no previous attempts to identify genotype–phenotype correlations in the area of oral surgery. Though Gürsoy et al. [12] performed an orodental examination and evaluation of the molecular characteristics of 17 patients diagnosed with NS, they hypothesized that mutations of the *PTPN11* gene may be associated with hypodontia. However, they based their conclusions on only two cases of hypodontia in patients with the same gene mutation. Though our study did not find this correlation with hypodontia, we found that seven patients out of seventeen with mutations in this gene presented supernumerary teeth.

Lutz et al. [11] described a 17-year-old male patient with mutations within the SOS1 gene, diagnosed with two mandibular odontomas, and it should be noted that their results are in compliance with ours. In our cohort, four patients with similar mutations were also diagnosed with mandibular compound odontomas. This is an interesting finding; although, due to the small sample size, the results should be considered with caution, especially as most research associates the existence of mutation within SOS1 with macrocephaly [13,14].

In the case of our patient with coexisting mutations in the PTPN11 and KRAS genes, the results obtained by Brasil et al. [15] confirm the possibility of such an occurrence. After retrieving the contact data of the patient’s guardians, we were able to contact his GDP, who informed us that it was a possible p.E69V mutation after consulting his notes. This would be in accordance with the fact that the patient was of a short stature and suffered from developmental delay and facial dysmorphia. It would also correspond with the results from PolyPhen-2 [16], as this variant is believed to be damaging. Regarding the results on the KRAS mutation, it was p.N85S. However, it is considered that the KRAS alternation does not possess any phenotypic effect, although it does influence developmental delay. This was the only case out of three patients with coexisting mutations that we could contact in order to broaden our interview.

Referring to oral and maxillofacial findings of other authors is difficult, mainly due to the fact that most research that might be considered as cohort studies [17,18] describe orthodontic features and present cephalometric measurements. Bagattoni et al. [18], who screened the orthopantomograms of 12 children with NS, mentioned only one patient with supernumerary primary upper lateral incisors; however, no information regarding molecular diagnosis was provided, even though the inclusion criteria included documented genetic diagnosis of NS.

The most significant conclusion of the present study is that, within the studied population, the incidence of bilateral and unilateral CGCG in the mandible was a characteristic feature of patients with mutations of *LZTR1*. Such lesions typically occur in the mandible during the first three decades of life, with a female predilection, which is supported by our findings. Our results contradict the theory of Luna et al. [19], who reported that multiple CGCGs in NS are associated with *PTPN11* or *SOS1* mutations. Similarly, Carcavilla et al. [20] support this conclusion; however, the research did not mention the correlation of the incidence of CGCG with mutations in genes encoding proteins of the RAS/MAPK kinase pathway. However, it attempted to determine other genotype–phenotype correlations, such as the definitive association of mutations of *PTPN11* and the presence of craniofacial anomalies, and the incidence of congenital cardiomyopathy with *SOS1* mutations.

Chinton et al. [21] studied four families and three sporadic NS cases with germline variants of the *LZTR1* gene, revealing that all patients had relative macrocephaly and visible facial dysmorphic changes; however, no oral examinations were performed in this study. Given that NS is associated with a higher incidence of cancer and the *LZTR1* gene acts as a tumor suppressor [22], it makes sense that somatic or germline loss of function variants in the *LZTR1* gene may be associated with increased incidence of CGCG. It is essential to increase awareness of the existing association between CGCG and NS in dental practitioners and pediatricians to aid in the timely diagnosis and treatment of these patients, as the occurrence of bilateral mandibular CGCG may lead to an erroneous diagnosis of cherubism [23,24]. In addition, giant-cell lesions may be aggressive, thus requiring prompt treatment to minimize morbidity [25,26].

In addition, prior to oral surgery, existing coagulation anomalies must be considered, as children with NS may suffer from various hematological disorders that affect primary and secondary hemostasis [27,28]. A study of 45 patients with NS by Yoshida et al. [29] revealed bleeding diathesis exclusively in patients with specific *PTPN11* mutations. Our research shows that, in the studied population, a history of hematologic anomalies was also present in children with *LZTR1* and *SOS1* mutations, contributing to peri- and post-operative surgical challenges. One of the restrictions of this retrospective study is that it presents the experience of a single research facility. However, it can be argued that, since our department is the leading oral and maxillofacial surgery provider for patients with rare diseases in the country, the participants represent a cross-section of the nationwide population and the obtained results can be comparable to those of other studies on children with NS, including those performed in multiple centers [10,18,19,30,31,32]. Another limitation is the small sample size, constituting only of patients who underwent surgical treatment. We strongly believe that it would be advantageous for other centers internationally to contribute their findings, if available, in order to gather more data.

In conclusion, although obtaining a detailed medical history is usually sufficient to provide dental care, this is not necessarily true for children with NS, where establishing a molecular diagnosis should become the standard of care. Differentiation between NS and other RASopathies poses a challenge, especially in the diagnosis of young infants or children, where even a detailed evaluation of medical and family history and examination focused on distinctive features is insufficient. Such children should be evaluated by clinical geneticists to interpret gene mutations, which will allow for the proper treatment of this patient population.

Although craniofacial features have been previously well described, the genotype–phenotype correlations in the field of oral surgery have not been analyzed in detail. We strongly believe that this study may enable better dental care in these patients.

## Figures and Tables

**Table 1:** Patient characteristics according to diagnosis.

Variable		*n*	%	Statistical Test Results *
Gender	Female	23	54.8	χ^2^(1) = 0.38; *p* = 0.54
	Male	19	45.2	
Diagnosis	Bilateral central giant-cell granulomas in mandible	8	19	χ^2^(6) = 15; *p* = 0.02
	Central giant-cell granuloma in mandible	6	14.3	
	Central giant-cell granuloma in maxilla	1	2.4	
	Mandibular compound odontoma	4	9.5	
	Maxillary compound odontoma	2	4.8	
	Retained deciduous teeth	10	23.8	
	Supernumerary teeth	11	26.2	

* Chi-square.

**Table 2:** Ages of the studied group.

Variable	M	Me	SD	Min	Max	Q1	Q3
Age	11.48	12	2.28	7	16	9.75	13

**Table 3:** Subject groups distinguished by genetic mutations.

Variable	*n*	%	Statistical Test Results *
*PTPN11*	17	40.5	χ^2^(1) = 1.52; *p* = 0.22
*SOS1*	7	16.7	χ^2^(1) = 18.67; *p* < 0.001
*RAF1*	4	9.5	χ^2^(1) = 25.72; *p* < 0.001
*KRAS*	4	9.5	χ^2^(1) = 25.72; *p* < 0.001
*BRAF*	0	0	-
*SHOC2*	0	0	-
*NRAS*	1	2.4	χ^2^(1) = 38.01; *p* < 0.001
*LZTR1*	12	28.6	χ^2^(1) = 7.71; *p* = 0.005
*CBL*	0	0	-

* Chi-square.

**Table 4:** Additional clinical parameters in the studied group.

Variable	*n*	%	Statistical Test Results *
History of hematologic anomalies	18	42.9	χ^2^(1) = 0.86; *p* = 0.36
Developmental delays	32	76.2	χ^2^(1) = 11.52; *p* = 0.001
Cardiac defects	32	76.2	χ^2^(1) = 11.52; *p* = 0.001
Craniofacial dysmorphia	42	100	-
Epilepsy	10	23.8	χ^2^(1) = 11.52; *p* = 0.001

* Chi-square.

**Table 5:** Incidence of individual genetic parameters in the studied group, divided according to clinical diagnosis.

Variable	Diagnosis														Statistical Test Results
	1		2		3		4		5		6		7		
	*n*	%	*n*	%	*n*	%	*n*	%	*n*	%	*n*	%	*n*	%	
*PTPN11*	0	0	3	50	0	0	0	0	1	50	6	60	7	63.6	**χ^2^(6) = 13.17; *p* = 0.04; V_cr_ = 0.56**
*SOS1*	0	0	0	0	1	100	4	100	2	100	0	0	0	0	**χ^2^(6) = 42; *p* < 0.001; V_cr_ = 1**
*RAF1*	0	0	0	0	0	0	0	0	0	0	1	10	3	27.3	χ^2^(6) = 6.24; *p* = 0.4
*KRAS*	0	0	0	0	0	0	0	0	0	0	4	40	0	0	**χ^2^(6) = 14.15; *p* = 0.03; V_cr_ = 0.58**
*BRAF*	0	0	0	0	0	0	0	0	0	0	0	0	0	0	-
*SHOC2*	0	0	0	0	0	0	0	0	0	0	0	0	0	0	-
*NRAS*	0	0	0	0	0	0	0	0	0	0	0	0	1	9.1	χ^2^(6) = 2.89; *p* = 0.82
*LZTR1*	8	100	4	66.7	0	0	0	0	0	0	0	0	0	0	**χ^2^(6) = 35.47; *p* < 0.001; V_cr_ = 0.92**
*CBL*	0	0	0	0	0	0	0	0	0	0	0	0	0	0	-

1—Bilateral central giant-cell granuloma (CGCG) in the mandible; 2—CGCG in the mandible; 3—CGCG in the maxilla; 4—mandibular compound odontoma; 5—maxillary compound odontoma; 6—over-retained deciduous teeth; 7—supernumerary teeth.

**Table 6:** Incidence of individual genetic parameters in the studied group, divided according to additional clinical findings.

Parameter	Variable									
	1		2		3		4		5	
	*n*	%	*n*	%	*n*	%	*n*	%	*n*	%
*PTPN11*	9	50	15	46.9	16	50	17	40.5	8	80
*SOS1*	4	22.2	6	18.8	7	21.9	7	16.7	1	10
*RAF1*	2	11.1	4	12.5	4	12.5	4	9.5	0	0
*KRAS*	0	0	4	12.5	4	12.5	4	9.5	2	20
*BRAF*	0	0	0	0	0	0	0	0	0	0
*SHOC2*	0	0	0	0	0	0	0	0	0	0
*NRAS*	0	0	1	3.1	0	0	1	2.4	0	0
*LZTR1*	5	27.8	4	12.5	4	12.5	12	28.6	1	10
*CBL*	0	0	0	0	0	0	0	0	0	0

1—History of hematologic anomalies; 2—developmental delays; 3—cardiac defects; 4—craniofacial dysmorphia; 5—epilepsy.

**Table 7:** Dependency of the sex of the studied group on the analyzed variables.

Parameter	Sex				Statistical Test Results *
	Female		Male		
	*n*	%	*n*	%	
PTPN11	11	47.8	6	31.6	χ^2^(1) = 1.14; *p* = 0.35
SOS1	3	13	4	21.1	χ^2^(1) = 0.86; *p* = 0.36
RAF1	2	8.7	2	10.5	χ^2^(1) = 0.04; *p* = 1
KRAS	1	4.3	3	15.8	χ^2^(1) = 1.58; *p* = 0.31
BRAF	0	0	0	0	-
SHOC2	0	0	0	0	-
NRAS	1	4.3	0	0	χ^2^(1) = 0.85; *p* = 1
LZTR1	7	30.4	5	26.3	χ^2^(1) = 0.09; *p* = 1
CBL	0	0	0	0	-
History of hematologic anomalies	13	56.5	5	26.3	χ^2^(1) = 3.88; *p* = 0.07
Developmental delays	18	78.3	14	73.7	χ^2^(1) = 0.12; *p* = 1
Cardiac defects	16	69.6	16	84.2	χ^2^(1) = 1.23; *p* = 0.31
Craniofacial dysmorphia	23	100	19	100	-
Epilepsy	7	30.4	3	15.8	χ^2^(1) = 1.23; *p* = 0.32

* Chi-square.

**Table 8:** Dependency of the age of the patients on individual genetic parameters and other variables in the studied group.

Parameter	Age
	Eta Coefficient
PTPN11	0.62
SOS1	0.36
RAF1	0.32
KRAS	0.52
BRAF	-
SHOC2	-
NRAS	0.32
LZTR1	**0.82**
CBL	-
History of hematologic anomalies	0.45
Developmental delays	**0.7**
Cardiac defects	0.62
Craniofacial dysmorphia	-
Epilepsy	0.51

## Data Availability

The data presented in this study are available upon request from the corresponding author. The data are not publicly available because the data are subject to further research.
